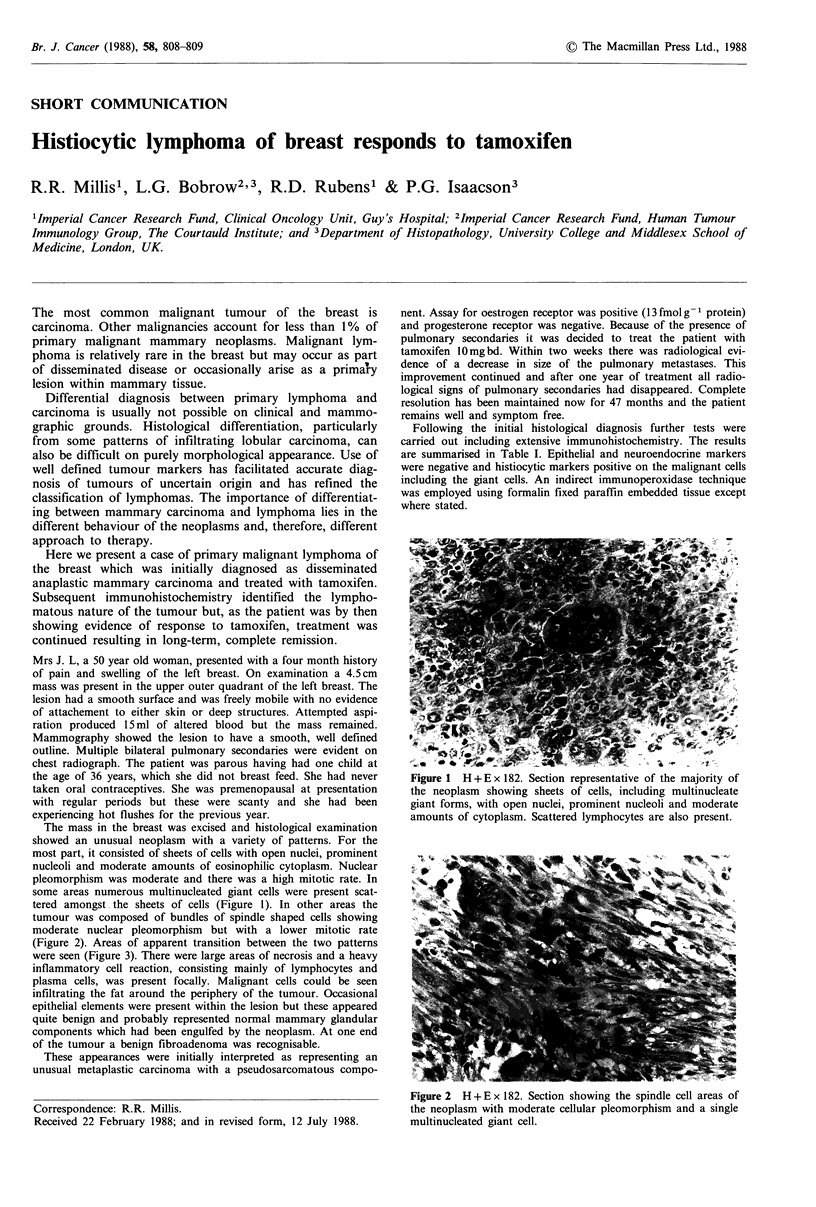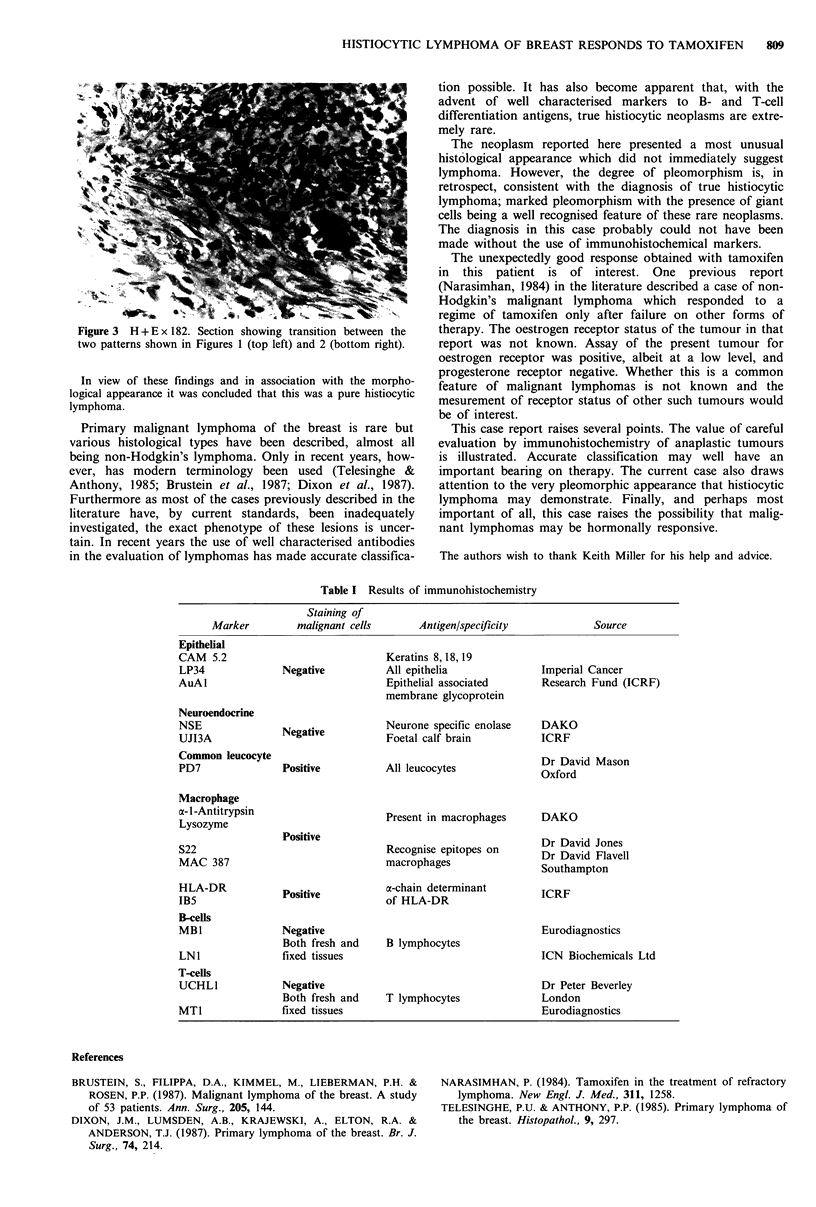# Histiocytic lymphoma of breast responds to tamoxifen.

**DOI:** 10.1038/bjc.1988.314

**Published:** 1988-12

**Authors:** R. R. Millis, L. G. Bobrow, R. D. Rubens, P. G. Isaacson

**Affiliations:** Imperial Cancer Research Fund, Guy's Hospital, London, UK.

## Abstract

**Images:**


Br. J. Cancer (1988), 58, 808–809 © The Macmillan Press Ltd., 1988

SHORT COMMUNICATION

### Histiocytic lymphoma of breast responds to tamoxifen

R.R. Millis[1], L.G. Bobrow[2,3], R.D. Rubens[1] & P.G. Isaacson[3]

[1]*Imperial Cancer Research Fund, Clinical Oncology Unit, Guy's Hospital;* [2]*Imperial Cancer Research Fund, Human Tumour Immunology Group, The Courtauld Institute; and* [3]*Department of Histopathology, University College and Middlesex School of Medicine, London, UK.*

The most common malignant tumour of the breast is carcinoma. Other malignancies account for less than 1% of primary malignant mammary neoplasms. Malignant lymphoma is relatively rare in the breast but may occur as part of disseminated disease or occasionally arise as a primary lesion within mammary tissue.

Differential diagnosis between primary lymphoma and carcinoma is usually not possible on clinical and mammographic grounds. Histological differentiation, particularly from some patterns of infiltrating lobular carcinoma, can also be difficult on purely morphological appearance. Use of well defined tumour markers has facilitated accurate diagnosis of tumours of uncertain origin and has refined the classification of lymphomas. The importance of differentiating between mammary carcinoma and lymphoma lies in the different behaviour of the neoplasms and, therefore, different approach to therapy.

Here we present a case of primary malignant lymphoma of the breast which was initially diagnosed as disseminated anaplastic mammary carcinoma and treated with tamoxifen. Subsequent immunohistochemistry identified the lymphomatous nature of the tumour but, as the patient was by then showing evidence of response to tamoxifen, treatment was continued resulting in long-term, complete remission.

Mrs J. L, a 50 year old woman, presented with a four month history of pain and swelling of the left breast. On examination a 4.5 cm mass was present in the upper outer quadrant of the left breast. The lesion had a smooth surface and was freely mobile with no evidence of attachement to either skin or deep structures. Attempted aspiration produced 15 ml of altered blood but the mass remained. Mammography showed the lesion to have a smooth, well defined outline. Multiple bilateral pulmonary secondaries were evident on chest radiograph. The patient was parous having had one child at the age of 36 years, which she did not breast feed. She had never taken oral contraceptives. She was premenopausal at presentation with regular periods but these were scanty and she had been experiencing hot flushes for the previous year.

The mass in the breast was excised and histological examination showed an unusual neoplasm with a variety of patterns. For the most part, it consisted of sheets of cells with open nuclei, prominent nucleoli and moderate amounts of eosinophilic cytoplasm. Nuclear pleomorphism was moderate and there was a high mitotic rate. In some areas numerous multinucleated giant cells were present scattered amongst the sheets of cells (Figure 1). In other areas the tumour was composed of bundles of spindle shaped cells showing moderate nuclear pleomorphism but with a lower mitotic rate (Figure 2). Areas of apparent transition between the two patterns were seen (Figure 3). There were large areas of necrosis and a heavy inflammatory cell reaction, consisting mainly of lymphocytes and plasma cells, was present focally. Malignant cells could be seen infiltrating the fat around the periphery of the tumour. Occasional epithelial elements were present within the lesion but these appeared quite benign and probably represented normal mammary glandular components which had been engulfed by the neoplasm. At one end of the tumour a benign fibroadenoma was recognisable.

These appearances were initially interpreted as representing an unusual metaplastic carcinoma with a pseudosarcomatous component. Assay for oestrogen receptor was positive (13 fmol $g^{-1}$ protein) and progesterone receptor was negative. Because of the presence of pulmonary secondaries it was decided to treat the patient with tamoxifen 10 mg bd. Within two weeks there was radiological evidence of a decrease in size of the pulmonary metastases. This improvement continued and after one year of treatment all radiological signs of pulmonary secondaries had disappeared. Complete resolution has been maintained now for 47 months and the patient remains well and symptom free.

Following the initial histological diagnosis further tests were carried out including extensive immunohistochemistry. The results are summarised in Table I. Epithelial and neuroendocrine markers were negative and histiocytic markers positive on the malignant cells including the giant cells. An indirect immunoperoxidase technique was employed using formalin fixed paraffin embedded tissue except where stated.

**Figure 1** H+E×182. Section representative of the majority of the neoplasm showing sheets of cells, including multinucleate giant forms, with open nuclei, prominent nucleoli and moderate amounts of cytoplasm. Scattered lymphocytes are also present.

**Figure 2** H+E×182. Section showing the spindle cell areas of the neoplasm with moderate cellular pleomorphism and a single multinucleated giant cell.

Correspondence: R.R. Millis.
Received 22 February 1988; and in revised form, 12 July 1988.

**Figure 3** H+E×182. Section showing transition between the two patterns shown in Figures 1 (top left) and 2 (bottom right).

In view of these findings and in association with the morphological appearance it was concluded that this was a pure histiocytic lymphoma.

Primary malignant lymphoma of the breast is rare but various histological types have been described, almost all being non-Hodgkin's lymphoma. Only in recent years, however, has modern terminology been used (Telesinghe & Anthony, 1985; Brustein *et al.*, 1987; Dixon *et al.*, 1987). Furthermore as most of the cases previously described in the literature have, by current standards, been inadequately investigated, the exact phenotype of these lesions is uncertain. In recent years the use of well characterised antibodies in the evaluation of lymphomas has made accurate classification possible. It has also become apparent that, with the advent of well characterised markers to B- and T-cell differentiation antigens, true histiocytic neoplasms are extremely rare.

The neoplasm reported here presented a most unusual histological appearance which did not immediately suggest lymphoma. However, the degree of pleomorphism is, in retrospect, consistent with the diagnosis of true histiocytic lymphoma; marked pleomorphism with the presence of giant cells being a well recognised feature of these rare neoplasms. The diagnosis in this case probably could not have been made without the use of immunohistochemical markers.

The unexpectedly good response obtained with tamoxifen in this patient is of interest. One previous report (Narasimhan, 1984) in the literature described a case of non-Hodgkin's malignant lymphoma which responded to a regime of tamoxifen only after failure on other forms of therapy. The oestrogen receptor status of the tumour in that report was not known. Assay of the present tumour for oestrogen receptor was positive, albeit at a low level, and progesterone receptor negative. Whether this is a common feature of malignant lymphomas is not known and the mesurement of receptor status of other such tumours would be of interest.

This case report raises several points. The value of careful evaluation by immunohistochemistry of anaplastic tumours is illustrated. Accurate classification may well have an important bearing on therapy. The current case also draws attention to the very pleomorphic appearance that histiocytic lymphoma may demonstrate. Finally, and perhaps most important of all, this case raises the possibility that malignant lymphomas may be hormonally responsive.

The authors wish to thank Keith Miller for his help and advice.

**Table I** Results of immunohistochemistry

| *Marker* | *Staining of malignant cells* | *Antigen/specificity* | *Source* |
|---|---|---|---|
| **Epithelial** | | | |
| CAM 5.2 | | Keratins 8, 18, 19 | |
| LP34 | **Negative** | All epithelia | Imperial Cancer |
| AuA1 | | Epithelial associated membrane glycoprotein | Research Fund (ICRF) |
| **Neuroendocrine** | | | |
| NSE | **Negative** | Neurone specific enolase | DAKO |
| UJ13A | | Foetal calf brain | ICRF |
| **Common leucocyte** | | | |
| PD7 | **Positive** | All leucocytes | Dr David Mason Oxford |
| **Macrophage** | | | |
| α-1-Antitrypsin | | Present in macrophages | DAKO |
| Lysozyme | **Positive** | | |
| S22 | | Recognise epitopes on macrophages | Dr David Jones |
| MAC 387 | | | Dr David Flavell Southampton |
| HLA-DR | **Positive** | α-chain determinant of HLA-DR | ICRF |
| IB5 | | | |
| **B-cells** | | | |
| MB1 | **Negative** | | Eurodiagnostics |
| | Both fresh and | B lymphocytes | |
| LN1 | fixed tissues | | ICN Biochemicals Ltd |
| **T-cells** | | | |
| UCHL1 | **Negative** | | Dr Peter Beverley |
| | Both fresh and | T lymphocytes | London |
| MT1 | fixed tissues | | Eurodiagnostics |

**References**

BRUSTEIN, S., FILIPPA, D.A., KIMMEL, M., LIEBERMAN, P.H. & ROSEN, P.P. (1987). Malignant lymphoma of the breast. A study of 53 patients. *Ann. Surg.*, **205,** 144.

DIXON, J.M., LUMSDEN, A.B., KRAJEWSKI, A., ELTON, R.A. & ANDERSON, T.J. (1987). Primary lymphoma of the breast. *Br. J. Surg.*, **74,** 214.

NARASIMHAN, P. (1984). Tamoxifen in the treatment of refractory lymphoma. *New Engl. J. Med.*, **311,** 1258.

TELESINGHE, P.U. & ANTHONY, P.P. (1985). Primary lymphoma of the breast. *Histopathol.*, **9,** 297.